# Median Nerve Diameter Ratio on Ultrasound as a Complementary Tool to Electrodiagnostic Testing in Carpal Tunnel Syndrome

**DOI:** 10.3390/diagnostics15192464

**Published:** 2025-09-26

**Authors:** Thorsten Lehnhardt, Christian Soost, Jan Adriaan Graw, Rene Burchard, Christopher Bliemel, Artur Barsumyan

**Affiliations:** 1 Faculty of Medicine, Philipps-University of Marburg, 35037 Marburg, Germany; 2 Faculty III: Statistic and Econometrics, University of Siegen, 57068 Siegen, Germany; 3 Department of Anesthesiology, Universitätsklinikum Erlangen, Friedrich-Alexander-Universität Erlangen-Nürnberg (FAU), 91054 Erlangen, Germany; 4 Department of Orthopedics and Traumatology, University Hospital of Giessen and Marburg, 35037 Marburg, Germany; 5 Department of Orthopedics and Trauma Surgery, Sports Medicine and Joint Centre, Lahn-Dill-Kliniken, 35683 Dillenburg, Germany; 6 Center for Trauma Surgery and Orthopaedics, Lahn-Dill-Kliniken, 35578 Wetzlar, Germany

**Keywords:** carpal tunnel syndrome, ultrasound, distal motor latency, median nerve

## Abstract

**Background**: Carpal tunnel syndrome is a common entrapment neuropathy of the upper limb that has a significant clinical and socioeconomic impact. Sonographic short-axis measurement of the median nerve cross-sectional area is a well-established complement to clinical examination and neurography. This study aimed to evaluate the correlation between the median nerve diameter ratio, distal motor latency, and sensory nerve conduction velocity. **Methods**: A total of 74 patients (94 hands and 93 evaluations) with carpal tunnel syndrome were examined. Ultrasound was performed using a Siemens Acuson X300 with a 10 MHz linear probe. Median nerve diameters proximal and within the carpal tunnel were measured in a longitudinal scan. The carpal tunnel ratio (proximal diameter/intratunnel diameter) was then calculated and correlated with distal motor latency. **Results**: No significant correlation was found between distal motor latency and the carpal tunnel ratio (r = 0.018, *p* = 0.8655). However, a weak, non-significant positive correlation was observed between sensory nerve conduction velocity and carpal tunnel ratio (r = 0.238, *p* = 0.326). **Conclusions**: Ultrasound cannot replace electrodiagnostic testing. In this cohort, no statistically significant association was observed between the carpal tunnel ratio and distal motor latency. While our findings do not support the use of this ultrasound parameter as a standalone diagnostic measure, sonographic assessment of the median nerve may still provide complementary information in selected clinical contexts.

## 1. Introduction

Carpal tunnel syndrome (CTS) is defined by compression of the median nerve in the carpal tunnel, a fibrocartilaginous passageway in the wrist. This compression is caused by a mismatch between the available space and its content. CTS is the most common peripheral nerve entrapment syndrome, and surgery for this condition is the second most common elective surgical procedure in Germany [1]. CTS occurs predominantly in females aged 40–70 years with an incidence rate of around 8–10%. It causes pain, numbness, and tingling in the hand (usually digitus I to III and radial side of digitus IV) and is an important cause of incapacity to work [2,3].

In most cases, the exact etiologic factors are unclear. Changes in the cross-section of these tendons due to structural changes in the microarchitecture caused by remodeling processes as a result of overuse or degeneration are cited as a possible cause [4]. In addition to mechanical factors, several comorbid conditions can increase the risk of CTS. These include diabetes mellitus, thyroid dysfunction, rheumatoid arthritis, obesity, pregnancy, and chronic kidney disease. All these conditions are associated with changes in connective tissue or fluid balance [4]. Such comorbidities contribute to the multifactorial etiology of CTS and must be considered when making a diagnosis and planning treatment [5].

The gold standard for the diagnosis of CTS so far is the clinical assessment in combination with electrodiagnostic testing [6,7,8]. In moderately advanced CTS, neurophysiological changes typically include prolongation of distal motor latency (DML) and reduction in sensory nerve conduction velocity (SNCV), often accompanied by reduced compound muscle action potential amplitude. Electroneurography is widely considered to be a reliable test, with reported sensitivities of 70–90% and specificities above 90% [9]. Electroneurography not only confirms the diagnosis but also provides information on disease severity, prognostic value, and indications for surgery [10]. A recent study also emphasized the added diagnostic value of electroneurography over clinical assessment alone, particularly in patients with atypical or borderline symptoms [5].

As a painless, non-invasive, inexpensive, and easily reproducible procedure, ultrasonography is certainly also attracting attention for the diagnosis of pathologies of the hand. So far, few studies have been able to reliably demonstrate an increase in the volume of the median nerve with ultrasound diagnostics [6,7,8]. Modern ultrasound devices enable precise visualization of the hand anatomy in the millimeter range, so that morphological nerve changes can be identified in a manner comparable to other advanced imaging modalities such as MRI [11].

Modern high-resolution soft tissue ultrasound has expanded the role of imaging in peripheral nerve diagnostics. It provides excellent visualization of many peripheral nerves and is already an established tool in the assessment of nerve entrapment and injury, e.g., cubital tunnel syndrome, brachial plexus injury, or axillary assessment for pain catheter placement [12]. Ultrasound can be used to assess not only the location and course of the nerve, but also its internal architecture [13].

The aim of this study was to evaluate the correlation between sonographic measurements of median nerve diameter at pre-defined anatomical levels proximal and within the carpal tunnel compared to DML and SNCV [14]. According to the study hypothesis, a higher ratio between the two sonographically measured diameters (proximal and within the carpal tunnel) would indicate a more pathological nerve change. In severe or long-standing cases, a relative reduction in the diameter of the nerve within the tunnel—the so-called ‘hourglass appearance’—may also occur, although in many cases the intratunnel diameter remains normal. By calculating the ratio between the measurements of nerve diameter proximal and within the carpal tunnel in a single sonographic plane, we aim to provide an indirect but accurate estimate of DML. Consequently, a direct correlation between this ratio and an increase in DML was expected.

## 2. Materials and Methods

### 2.1. Study Population

This retrospective study was conducted using data collected from patients diagnosed with CTS. This study included 74 patients diagnosed with CTS. The study population was predominantly female (70% of participants were women, with 53 women and 21 men). The mean age was 56.5 years (range 19–84 years), with a median of 56 years. The age distribution showed that 9.3% of patients were in the 30–40 age range and only 12.1% were in the 60–70 age range. The mean body mass index (BMI) was 28.6 kg/m^2^. The mean symptom duration was 16 months. Bilateral symptoms were present in 38 patients. Among them, 38 presented with bilateral symptoms. However, not all symptomatic hands could be examined due to incomplete data. In total, 94 hands were included in the analysis, of which 93 had complete ultrasound and electrodiagnostic evaluations. One hand was excluded because of missing electrodiagnostic data. All sonographic assessments were performed by the same physician to minimize examiner-dependent variability. The examiner holds the German Society for Ultrasound in Medicine (DEGUM) certification in ultrasonography, which is internationally recognized as evidence of advanced expertise and quality standards in diagnostic ultrasound.

Inclusion criteria were age ≥ 18 years; clinically confirmed CTS symptoms (e.g., nocturnal paresthesia, numbness, or pain in the distribution of the median nerve, weakness or clumsiness of the hand, and positive provocative tests such as Tinel’s or Phalen’s sign); distal motor latency > 4.2 ms as documented by a neurologist [14]; and absence of acute nerve injuries causing neurological deficits. Only patients with both clinical symptoms and electrophysiological evidence of median nerve compression were included. Patients who presented with typical CTS symptoms but had negative findings on nerve conduction studies were excluded. Comorbidities and current medication use were documented but did not affect eligibility.

This study was conducted in accordance with the Declaration of Helsinki (2013) and the ethical board of Philipps-University of Marburg approved the study (23-152 RS), approved date: 19 June 2023. The study was not registered in a database. The data are available from the corresponding author upon reasonable request.

### 2.2. Ultrasound Technique

**Figure 1:**
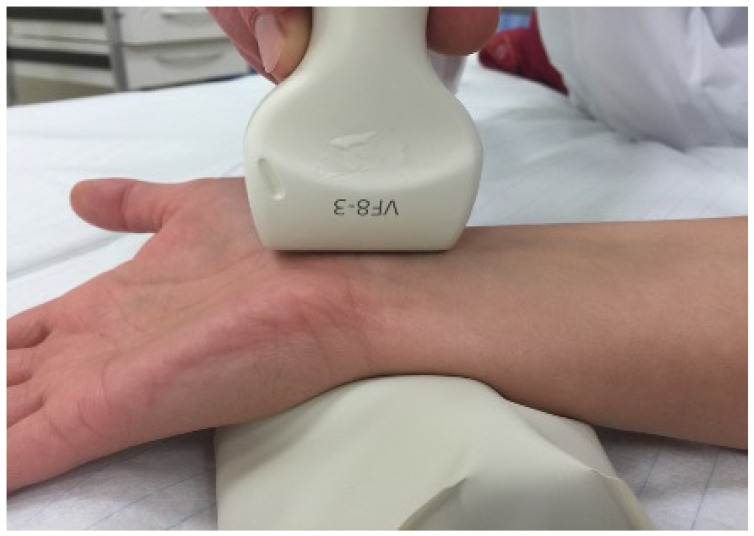
Positioning of the probe over the transverse carpal ligament.

All ultrasound examinations were performed with an Acuson X300 (Siemens Medical Solutions USA, Inc., Malvern, PA, USA), which was equipped with a linear transducer and was routinely available in our clinic. The system operated in a mode preset by the manufacturer and optimized for carpal tunnel examination at a frequency of 10 MHz. No changes were made to the device for the purposes of this study. The sonographic examination was performed in a single longitudinal plane to allow visualization and measurement of the diameter of the median nerve, both proximally and within the carpal tunnel, in a continuous setting. The probe was positioned over the transverse carpal ligament so that both measurement points could be acquired simultaneously (Figure 1).

This approach was intended to detect compression effects due to changes in nerve diameter and echo texture. The ultrasound probe was positioned transversely at the level of the carpal tunnel inlet, and the nerve was aligned to optimize visualization of its cross-sectional profile. Care was taken to capture the maximum anterior–posterior (dorsal-volar) diameter of the median nerve in this plane, although in some patients—particularly those with larger hands—complete visualization across the wrist could be challenging. The ‘one-slice’ longitudinal approach differs from the standard cross-sectional area (CSA) method recommended in current guidelines. Although it was chosen to simplify the examination, it could limit comparability with previous studies and may result in three-dimensional volumetric changes to the nerve being overlooked.

### 2.3. Measurement of Median Nerve Diameters

**Figure 2:**
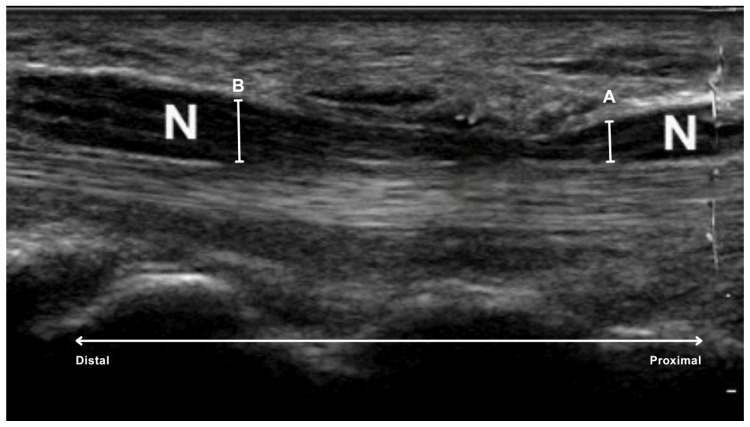
Sonographic measurement sites of the median nerve (N) in carpal tunnel syndrome. A, measuring point at the level of the first carpal row; B, Measuring point under the flexor retinaculum.

To quantify the degree of nerve compression, the ratio of the sonographically measured diameters of the median nerve was calculated: the proximal diameter (mm) divided by the diameter within the carpal tunnel (mm). The measurements were taken on frozen images using the caliper built into the system (Figure 2).

Two specific locations were defined:

1. proximal to the carpal tunnel, at the level of the first carpal row, specifically over the os lunatum, the os triquetrum and the base of the os scaphoideum (A in Figure 2).
2. inside the carpal tunnel, centrally under the flexor retinaculum, at the level of the tuberosity of the scaphoid and trapezium bones on the radial side and the hamulus of the hamate and pisiform bones on the ulnar side (B in Figure 2).

The carpal tunnel ratio served as a single numerical expression for the change in caliber of the nerve and was then compared to the neurographically measured DML (ms). The aim was to create a practical, easy-to-use index reflecting the severity of the structural nerve change. The greater the ratio based solely on sagittal diameter measurements, the greater the suspected pathologic change; cross-sectional area was not considered. To test this relationship, the Bravais-Pearson correlation coefficient was calculated.

Electroneurography was performed on all patients by board-certified neurologists, in accordance with standard procedures. For motor conduction studies, stimulation of the median nerve was performed at the wrist, with recordings obtained from the abductor pollicis brevis muscle. DML was defined as the time in milliseconds from stimulation to the onset of the compound muscle action potential; values exceeding 4.2 ms were considered pathological [14]. Sensory conduction studies were performed orthodromically, with stimulation of the index finger and recording at the wrist. SNCV was calculated by dividing the conduction distance by onset latency, using laboratory-specific normative cut-offs. All studies were performed in a temperature-controlled setting (skin temperature ≥ 32 °C) to ensure reproducibility.

### 2.4. Statistical Analysis

Data analysis was performed using R (R Core Team (2021). R: A language and environment for statistical computing. R Foundation for Statistical Computing, Vienna, Austria.). To assess the relationship between the sonographic ratio and distal motor latency, bivariate correlations were analyzed using Bravais–Pearson correlation coefficients. A sensitivity power analysis (G*Power 3.1) showed that with *n* = 93, α = 0.05, and power = 0.80, the minimum detectable correlation is r = 0.29 (r = 0.31 with Bonferroni correction. The statistical significance was set at *p* < 0.05. The statistical significance was set at *p* < 0.05.

## 3. Results

### 3.1. Correlation Between Carpal Tunnel Ratio and Prolonged Distal Motor Latency

The results of the correlation analysis showed no significant correlation between the sonographic carpal tunnel ratio and DML, thus failing to confirm the original hypothesis (r = 0.018, *p* = 0.8655).

**Figure 3:**
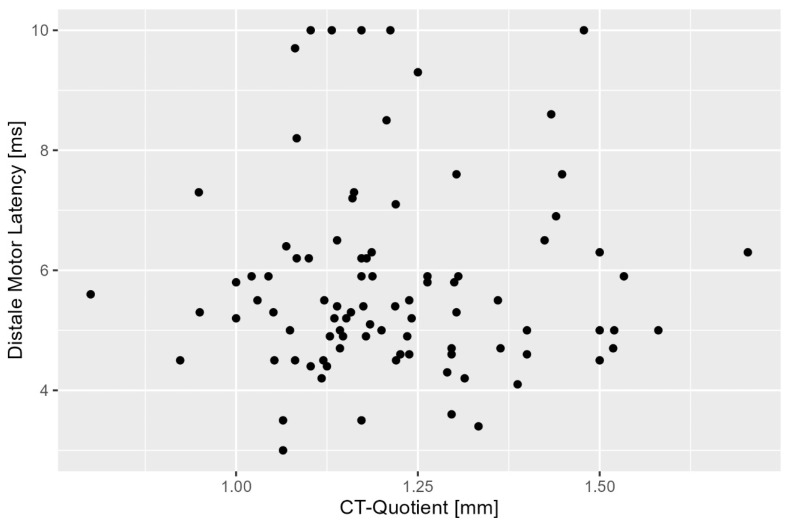
Scatterplot between carpal tunnel (CT) ratio and distal motor latency.

This result suggests that the sonographic carpal tunnel ratio does not reflect the degree of neurophysiologic impairment as measured by distal motor latency (Figure 3).

### 3.2. Correlation Between CT Ratio and Sensory Nerve Conduction Velocity

**Figure 4:**
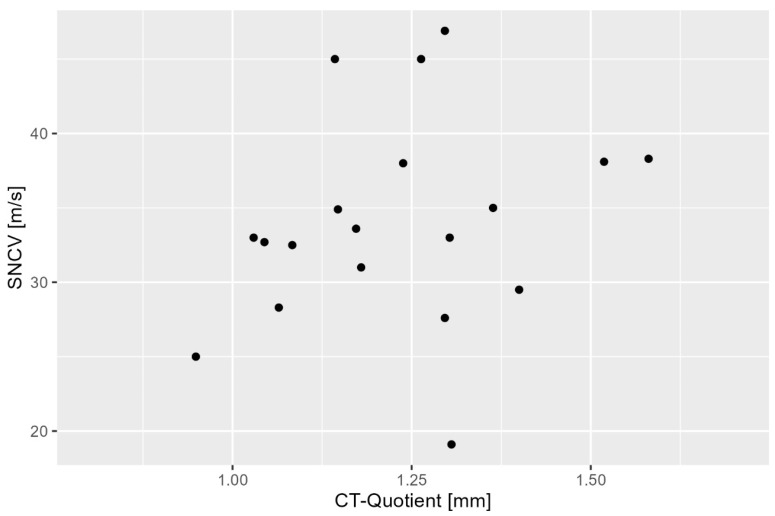
Scatterplot between carpal tunnel (CT) ratio and SNCV. SNCV—sensory nerve conduction velocity.

The relationship between ultrasound measurements and sensory nerve conduction velocity (SNCV) was evaluated to determine whether the sonographic ratio could reflect sensory nerve function (Figure 4). SNCV testing was performed in a subset of the study population, consisting of 19 randomly selected patients, resulting in data from 19 hands.

The analysis revealed a weak positive but non-significant correlation between the sensory nerve conduction velocity and the carpal tunnel ratio (r = 0.238, *p* = 0.326). Thus, no statistically significant correlation was found, and the ultrasound-based measurements did not provide a reliable assessment of sensory nerve function in CTS.

## 4. Discussion

This study investigated whether sonographic measurements of the median nerve diameter at defined anatomical levels proximal and within the carpal tunnel correlate with electrodiagnostic parameters, specifically DML and SNCV. The analyses showed no correlation between the diameter of the median nerve in front of the CT and in the CT itself in terms of distal motor latency. Therefore, ultrasonography does not appear to be an alternative stand-alone diagnostic tool for the detection of CTS.

Current guidelines such as the German S3 guideline for hand surgery, neurosurgery, neurology and orthopedics and the guideline of the American Association of Electrodiagnostic Medicine (AAEM) define a structured diagnostic pathway to assess CTS [1,15]. After medical history taking and clinical assessment, electrodiagnostic testing is recommended as the gold standard for confirming the diagnosis of CTS [16]. The most common electrodiagnostic tests are SNCV and DML, which are considered reliable and mandatory for diagnosis by the professional societies [17,18,19].

In contrast to the previous study, where cross-sectional measurements were taken at anatomically defined points, this study used a longitudinal “one-slice” approach to assess nerve compression based on diameter differences alone [20]. While longitudinal imaging is a standard procedure for assessing nerve course, documenting diameter changes at two pre-defined locations with a single probe setting could further standardize and simplify the procedure [21]. The aim was to investigate whether such a protocol could still yield diagnostically relevant correlations with DML. However, the results of this study showed that this method neither reliably predicts DML nor correlates with SNCV. Potential reasons could be the individual variability of nerve remodeling and a significant methodological limitation such as the lack of a third spatial dimension in the 2D imaging technique. The nerve might adapt by changing its volume in depth (outside the image plane), which cannot be captured in the sagittal plane alone [22]. Consequently, volume changes without diameter changes in the image plane remain undetected, leading to a potential systematic measurement error.

In addition, measurement of the cross-sectional area is generally considered more accurate and recommended in the current literature and professional guidelines but requires two different ultrasound slices and accurate anatomic identification [23,24,25]. Any deviation in probe placement can lead to a significant measurement error. The “one-slice” method could simplify the procedure, but however at the cost of potentially missing important structural changes.

The present study found no significant correlation between the diameter ratio of the median nerve (proximal versus inside the carpal tunnel) and DML. Therefore, the hypothesis that prolonged DML can be predicted based on sonographic measurements alone must be rejected. This finding is consistent with previous studies that used the cross-sectional area rather than the diameter as a comparator and also failed to demonstrate a reliable correlation with DML. While these studies confirmed the presence of CTS by sonographic findings, the results did not support the quantitative prediction of electrodiagnostic parameters [26,27].

In the present study, measurements were taken at two anatomically defined levels: first, proximal to the tunnel at the level of the proximal carpal bones and, second, below the transverse carpal ligament at the level of the capitate bone [28,29]. Based on these measurements, a mean diameter ratio of 1.21 was found. However, subsequent analysis revealed no statistically significant correlation between this ratio and prolonged DML. Therefore, despite the methodological emphasis on simplicity and clinical feasibility, the longitudinal “one-slice” approach using the diameter ratio does not provide reliable evidence of neurophysiological impairment in CTS. Ideally, this approach would allow the determination of DML based on ultrasound data alone, which would significantly reduce the need for electrodiagnostic nerve conduction studies, provided that the sonographic measurements have a sufficiently low error rate [30].

The negative results of this study highlight that the proposed sonographic ratio cannot replace electrophysiological testing. In clinical terms, this means that ultrasound in this simplified form cannot quantify functional impairment of the median nerve and therefore electrodiagnostic confirmation remains necessary. Furthermore, they highlight that negative correlations must be interpreted as a clear limitation of the method’s diagnostic utility. Although the one-slice longitudinal ratio was designed to streamline the examination process, the lack of correlation with DML or SNCV suggests that this methodology does not offer any diagnostic advantages. While there is a broad consensus that ultrasound is effective at detecting the structural consequences of CTS, it is less reliable at grading severity [31]. Conversely, conventional ultrasound of the CSA at the carpal tunnel inlet has been shown to distinguish CTS from healthy controls with moderate sensitivity and specificity. Combining inlet and outlet measurements can improve sensitivity by up to 20% [32]. Therefore, compared to CSA-based methods, our approach appears to be less reliable and cannot currently be recommended for clinical use beyond exploratory evaluation in other clinical studies [33,34].

### Limitations

Although our study had adequate power to detect small-to-moderate correlations (r ≥ 0.29), weaker associations may not have been detectable. Other limitations include the retrospective, single-center design, which carries an inherent risk of selection bias, and the absence of a control group, which restricts the study’s external validity. Furthermore, analysis of sensory nerve conduction velocity was limited to a smaller subset of patients (n = 19 hands), which reduces the reliability of conclusions drawn for this parameter. Furthermore, potential confounders such as age, sex, BMI, and symptom duration were not included in the analysis. Further concerns relate to the known higher sensitivity of nerve conduction studies compared to sonography. The three-dimensional changes in the nerve that cannot be captured with two-dimensional longitudinal studies are another potential source of error, which is usually mitigated by cross-sectional measurements at two locations. Relying solely on sagittal diameters carries the risk of overlooking volumetric nerve remodeling, which could introduce a systematic error. Although intra- and inter-observer reliability was not assessed because all examinations were performed retrospectively by a single experienced examiner, this remains an important factor for future validation. Operator dependency leads to further variability, as ultrasound examination requires a high level of experience and skill, which is reflected in well-documented discrepancies between examiners in different anatomical regions.

## 5. Conclusions

Although sonography might have a role in the diagnosis of CTS, especially with the simplified “one-slice” protocol based on the ratio of longitudinal diameters, it cannot replace electrodiagnostic testing. The results of this study reinforce the view that ultrasound should be used as a complementary tool to support clinical findings and electrodiagnostic data, but not as a stand-alone diagnostic method. Further studies using three-dimensional imaging or advanced cross-sectional protocols may help to refine the role of ultrasound in the diagnosis of CTS and improve its correlation with functional nerve parameters.

## Data Availability

Raw data supporting the conclusions of this article will be made available by the authors upon reasonable request, without undue reservation.

## Abbreviations

The following abbreviations are used in this manuscript:

CTS	Carpal tunnel syndrome
CSA	Cross-sectional area
DML	Distal motor latency
SNCV	Sensory nerve conduction velocity
